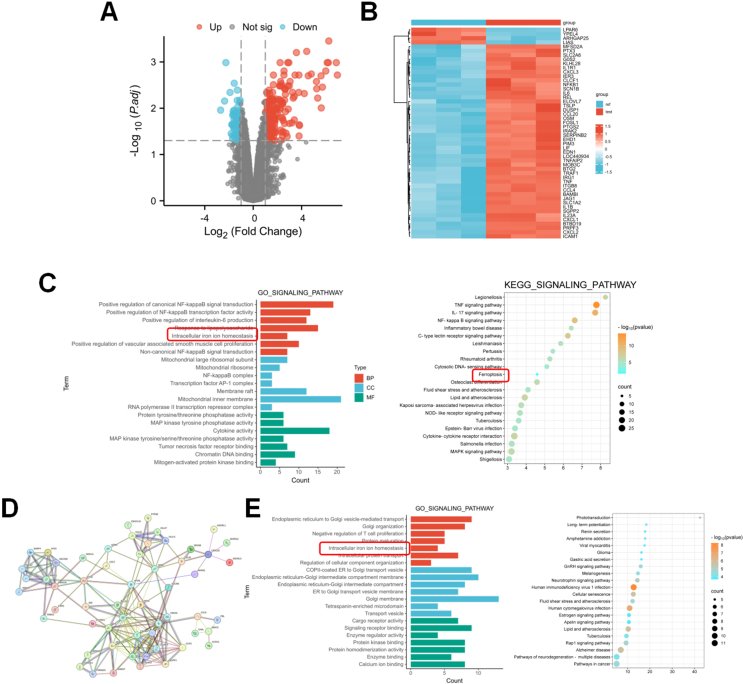# Single-cell transcriptomics unveils iron dysregulation in macrophages: Implicated genes in periodontitis pathogenesis identified via Mendelian randomization

**DOI:** 10.1016/j.gendis.2025.101544

**Published:** 2025-01-22

**Authors:** Kun Wang, Qingyue Xiao, Xinyu Liu, Wenjie Zhong, Ping He, Jingsong Ren, Jinda Li, Jie Zhou, Yan Bai, Xiang Gao

**Affiliations:** a College of Stomatology, Chongqing Medical University, Chongqing 401147, China; b Chongqing Key Laboratory of Oral Diseases, Chongqing 401147, China; c Chongqing Municipal Key Laboratory of Oral Biomedical Engineering of Higher Education, Chongqing 401147, China; d Department of Stomatology, Dazhou Central Hospital, Dazhou, Sichuan 635000, China; e School of Pharmacy, Chongqing Medical University, Chongqing 400016, China

Periodontitis, a chronic inflammatory condition, is one of the leading causes of tooth loss globally, contributing significantly to the burden of oral diseases. Its pathogenesis involves a multifaceted interaction between microbial dysbiosis and host immune responses, where macrophages are pivotal in regulating inflammation and tissue remodeling. These immune cells exhibit dual functionality, contributing to both protective immunity and pathological responses, thus underscoring their importance in the progression of periodontitis. Recent research demonstrates that iron metabolism, a critical regulator of immune cell function, strongly influences macrophage activity and may worsen the inflammatory microenvironment of periodontitis. Nevertheless, the molecular mechanisms that link iron homeostasis with macrophage-mediated periodontal destruction are not fully understood.^1^

Iron is critical in many physiological processes, like immune regulation, but iron dysregulation is linked to worsening inflammation and increased oxidative stress.^2^ There is clinical evidence for a paradoxical relationship between iron deficiency anemia and iron overload and the degree of periodontitis. The duality of the findings indicates that iron metabolism not only reflects systemic health but actively supports disease progression through modulating macrophage polarization, cytokine secretion, and oxidative stress, which are central drivers of the underlying immune context of periodontal tissues. However, these molecular pathways are not fully understood and their knowledge is crucial for identifying targets to restore immune balance and prevent tissue destruction in periodontitis.^3^

To gain further insight into this question, single-cell transcriptomics (single-cell RNA sequencing) was combined with Mendelian randomization to examine whether iron metabolism-related genes have a role in macrophage-driven inflammation. High-resolution profiling of immune cell heterogeneity in periodontal tissues is made possible by single-cell RNA sequencing, with information about macrophage subpopulations and function. Finding causal relationships, while minimizing confounding factors, is what Mendelian randomization does through its leverage for genetic variants. We obtained gene expression data from the Gene Expression Omnibus aimed at single-cell RNA sequencing datasets. To identify genetic variants associated with iron metabolism, the eQTLGen consortium coordinated and integrated genetic data from the eQTLGen consortium and from genome-wide association studies from the FinnGen project. ClusterProfiler was used to analyze differentially expressed genes in macrophages enriched for iron-related pathways and Mendelian randomization was used to evaluate causal links.

Using the GSE171213 dataset, 23 distinct immune cell clusters were identified (Fig. 1A), and these were annotated based on specific marker genes (Fig. 1B). A heatmap analysis validated marker specificity, providing molecular insights into their roles (Fig. 1C). Among the identified clusters, macrophages emerged as the central immune cell type significantly associated with the inflammatory microenvironment characteristic of periodontitis. Comparative analysis of cell-type proportions revealed notable differences between periodontitis and control groups, highlighting the altered immune landscape in disease states (Fig. 1D). Differential gene expression analysis of macrophages identified 419 significantly altered genes, many of which were enriched in pathways linked to iron metabolism (Fig. 1E).

**Figure 1:**
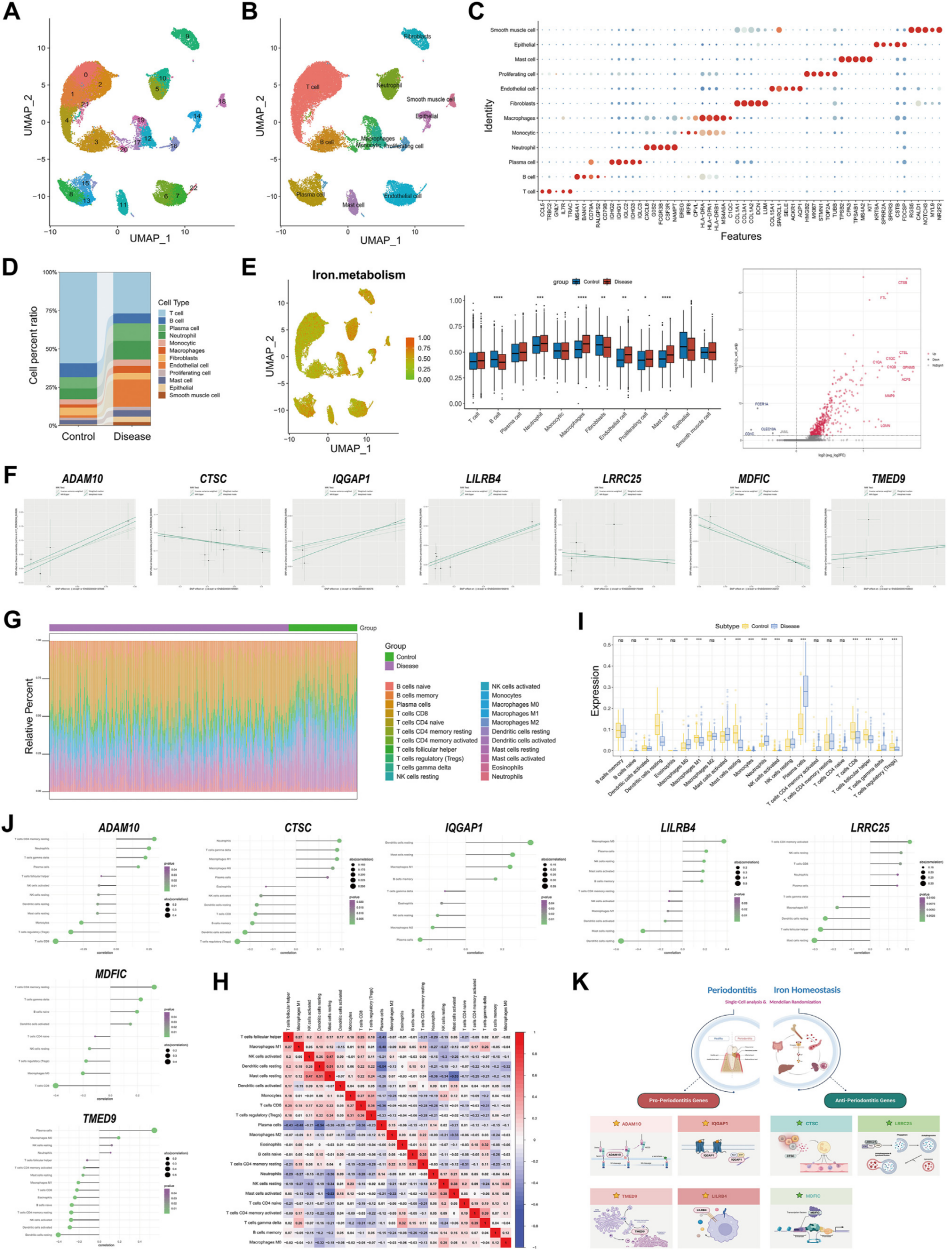
Integrated genomic and cellular analysis of periodontitis and iron metabolism dynamics. **(A)** UMAP analysis of cellular clusters. **(B)** Annotation of 12 cell types. **(C)** Doplot of cell marker associations. **(D)** Cell type distribution comparison. **(E)** Volcano plot of gene expression differences between groups, color-coded for down- and up-regulation. **(F)** Mendelian randomization analysis scatter plots for key genes, differentiated by statistical methods. **(G)** Proportions of 22 immune subpopulations. **(H)** Correlation analysis of immune subpopulations, color-coded for positive and negative relationships. **(I)** Immune cell distribution differences between groups. **(J)** Key gene-immune cell correlation. **(K)** Schematic of periodontitis-iron metabolism interplay and key gene roles.

Further analysis through Mendelian randomization identified seven genes (ADAM10, CTSC, IQGAP1, LILRB4, LRRC25, MDFIC, and TMED9) as causally associated with periodontitis (Fig. 1F). STRING-based protein-protein interaction analysis revealed functional connections among these genes, implicating pathways involving intracellular iron regulation, immune responses, and inflammation (Fig. S9D, E). To investigate immune interactions influenced by these key genes, immune infiltration patterns and cell-cell correlations were examined. Significant variations in immune cell populations, including macrophages M0 and M1, neutrophils, T cells (CD4^+^ memory resting, CD8^+^, and regulatory T cells), dendritic cells, and natural killer cells, were observed across disease states (Fig. 1G). Correlation analysis further highlighted complex relationships among these immune cell populations, reflecting their dynamic interplay in shaping the inflammatory microenvironment of periodontitis (Fig. 1H).

To explore the functional relevance of the seven genes in immune responses, gene-immune cell correlation analysis was conducted. ADAM10 (ADAM metallopeptidase domain 10) exhibited positive correlations with CD4^+^ memory resting T cells and neutrophils while showing negative associations with regulatory T cells and CD8^+^ T cells. Similarly, CTSC (cathepsin C) demonstrated positive correlations with M1 macrophages and neutrophils, underscoring its involvement in inflammatory pathways. IQGAP1 (IQ motif containing GTPase activating protein 1) displayed mixed correlations with dendritic cells and macrophages. These findings emphasize the regulatory roles of these genes in macrophage-driven inflammation and immune modulation within the periodontal microenvironment (Fig. 1I, J).

To validate these findings, the GSE24897 dataset, which profiles macrophages exposed to *P. gingivalis* (a key periodontal pathogen), was integrated. Differential expression and enrichment analyses revealed significant alterations in iron metabolism-related pathways in macrophages, supporting the hypothesis that iron dysregulation drives immune-inflammatory imbalances in periodontitis (Fig. S9A–C). Functional enrichment analyses using Gene Ontology (GO) and Kyoto Encyclopedia of Genes and Genomes (KEGG) pathway analysis further demonstrated the involvement of these genes in processes related to iron homeostasis, oxidative stress, and immune regulation (Fig. S2, S5). Notably, ADAM10, LILRB4 (leukocyte immunoglobulin-like receptor B4), and TMED9 (transmembrane P24 trafficking protein 9) were directly annotated with iron-related functions in the GeneCards database, reinforcing their roles in iron metabolism.

In the supplementary materials, comprehensive methodological and analytical insights are provided in Figure S1–8, and the steps taken to ensure the robustness of single-cell RNA sequencing data through quality control are illustrated in Figure S1. The differential gene enrichment within immune-related pathways is highlighted (Fig. S2). The seven gene-causal relationships are demonstrated to be robust through leave-one-out sensitivity analyses (Fig. S3). Key genes are shown to correlate with various immune factors, including chemokines and immunomodulatory agents (Fig. S4). The roles of these genes in immune pathways, including NF-kB (nuclear factor kappa B) and ferroptosis, and their alignment with iron metabolism and inflammation are emphasized (Fig. S5, S6). Gene Set Variation Analysis (GSVA) pathway analyses are presented (Fig. S7). Transcriptional regulatory networks and single-cell expression profiles of these genes are visualized, providing insights into their functional relevance (Fig. S8).

Further mechanistic insights were obtained by exploring specific evidence linking ADAM10 and LILRB4 to iron metabolism. ADAM10, identified as an interactor of the amyloid precursor protein (APP), plays a pivotal role in modulating iron homeostasis. APP is extensively studied for its role in regulating ferritin mRNA translation and mitigating oxidative damage.^4^ Similarly, LILRB4 has been implicated in iron metabolism through its downstream effector, PTPN6 (protein tyrosine phosphatase non-receptor type 6), which is linked to ferroptosis and disrupted iron homeostasis.^5^ Together, these findings reinforce the hypothesis that iron metabolism and macrophage-driven inflammation are intricately linked in periodontitis, mediated by the regulatory functions of these key genes.

Functional interactions among the identified genes and their connections to iron metabolism and immune modulation are substantiated by the protein-protein interaction network (Fig. S9D, E). These supplementary analyses collectively validate the robustness of the findings and emphasize the biological significance of the identified genes in the pathogenesis of periodontitis (Fig. 1K).

Overall, this study demonstrates convincing evidence for a new connection between macrophage dysfunction, iron dysregulation, and periodontitis pathogenesis. We provide strong evidence for seven genes in macrophage-driven immune responses and their roles in periodontal microenvironments based on single-cell RNA sequencing and Mendelian randomization. These results establish a basis for further research aiming to target therapeutic interventions against macrophage-driven inflammation and iron metabolism in periodontitis.

## Funding

This study was supported by grants from the Chongqing Young and Middle-aged Medical High-end Talent Studio (2022CQBSHTB3083) , the Program for Youth Innovation in Future Medicine of Chongqing Medical University (No. W0055) and the Chongqing Municipal Postdoctoral Science Foundation Project (CSTB2023NSCQ-BHX0033).

## CRediT authorship contribution statement

**Kun Wang:** Writing – original draft, Validation, Software, Methodology, Investigation, Formal analysis, Data curation, Conceptualization. **Qingyue Xiao:** Investigation, Data curation. **Xinyu Liu:** Investigation, Data curation. **Wenjie Zhong:** Investigation, Data curation. **Ping He:** Data curation. **Jingsong Ren:** Data curation. **Jinda Li:** Data curation. **Jie Zhou:** Investigation, Data curation. **Yan Bai:** Data curation. **Xiang Gao:** Writing – review & editing, Supervision, Resources, Project administration, Funding acquisition, Conceptualization.

## Conflict of interests

All authors declared no competing interests.

## Figures and Tables

**Fig. S1:**
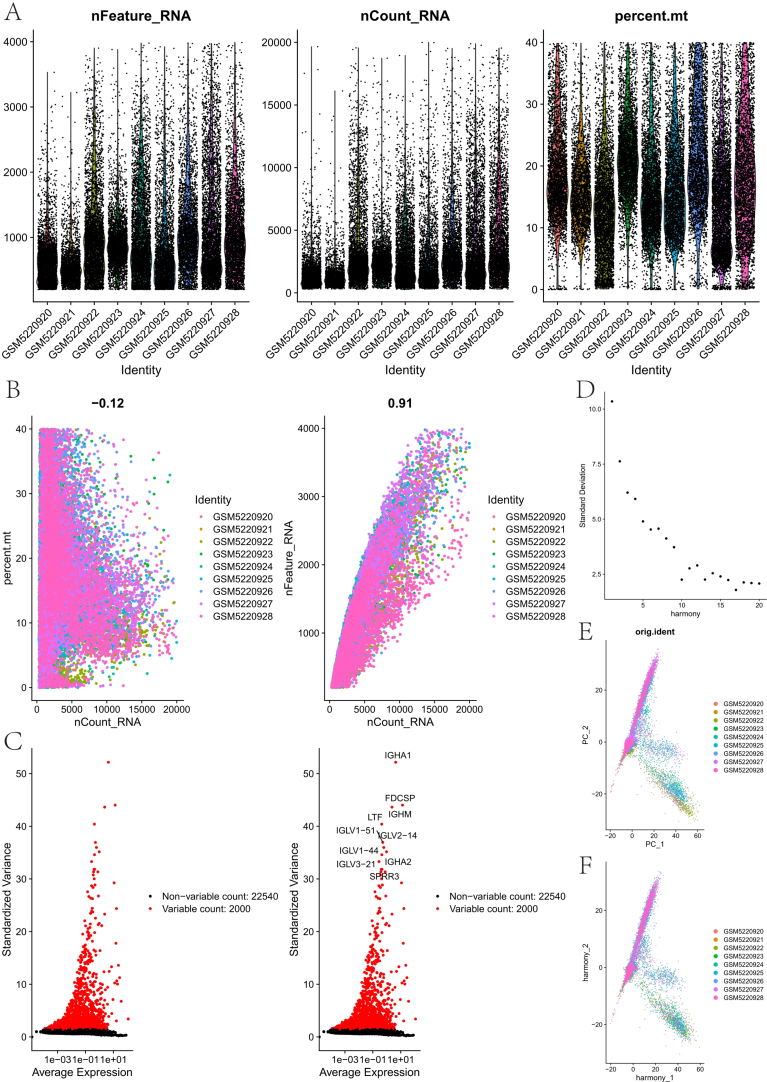


**Fig. S2:**
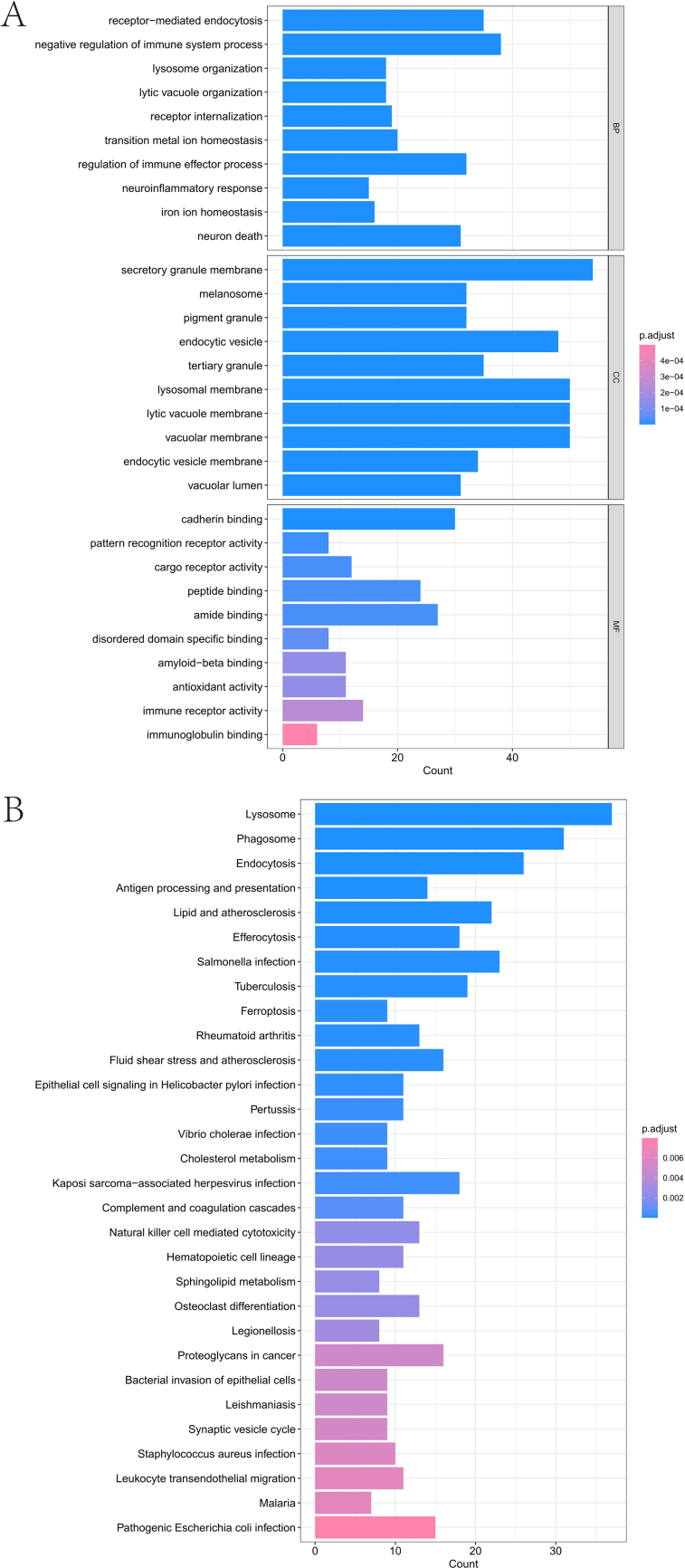


**Fig. S3:**
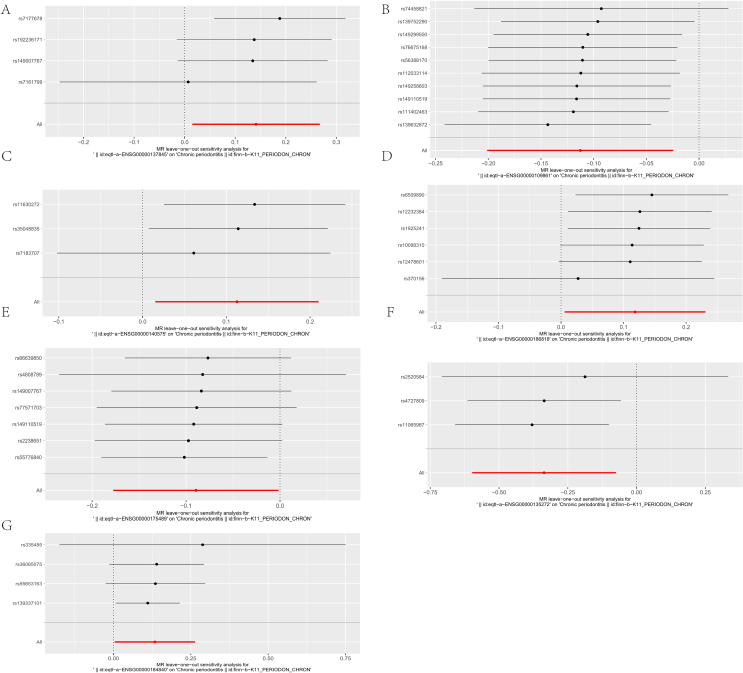


**Fig. S4:**
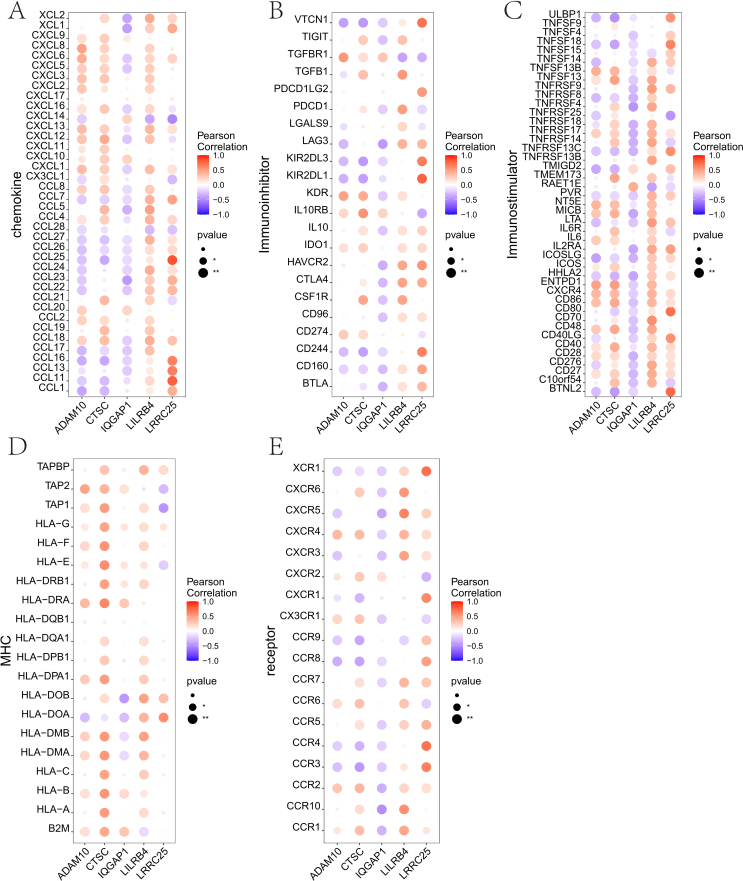


**Fig. S5:**
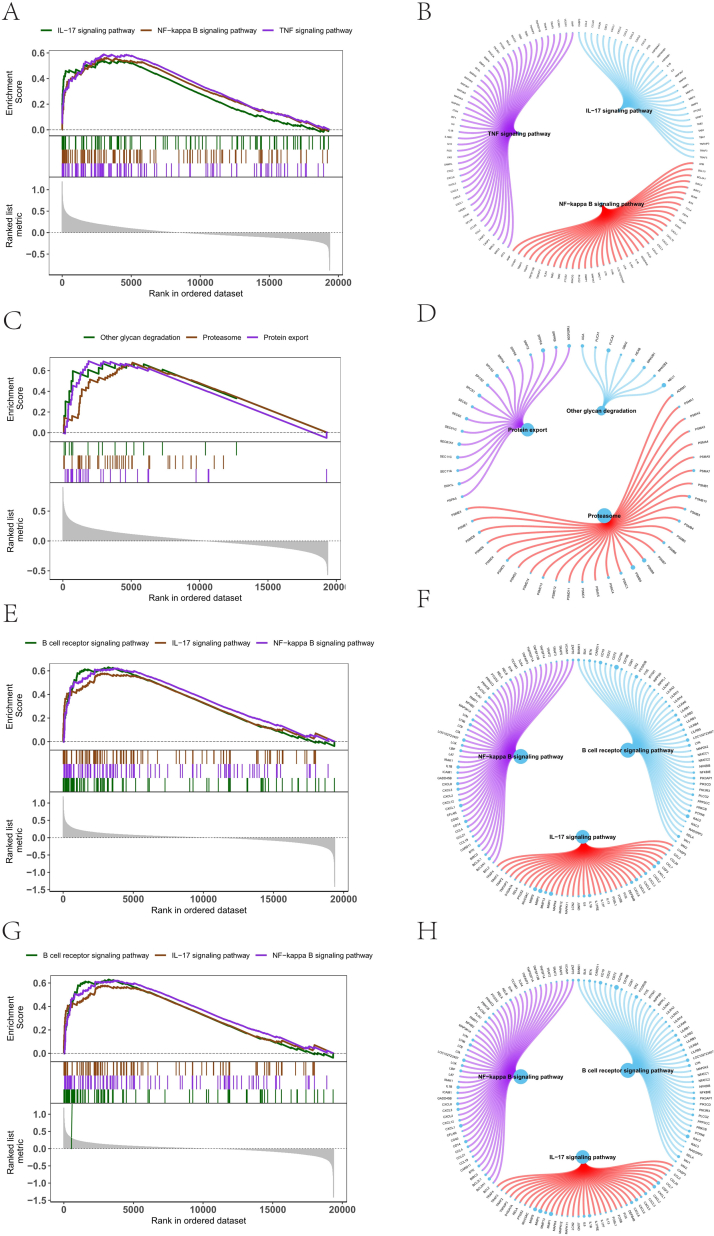


**Fig. S6:**
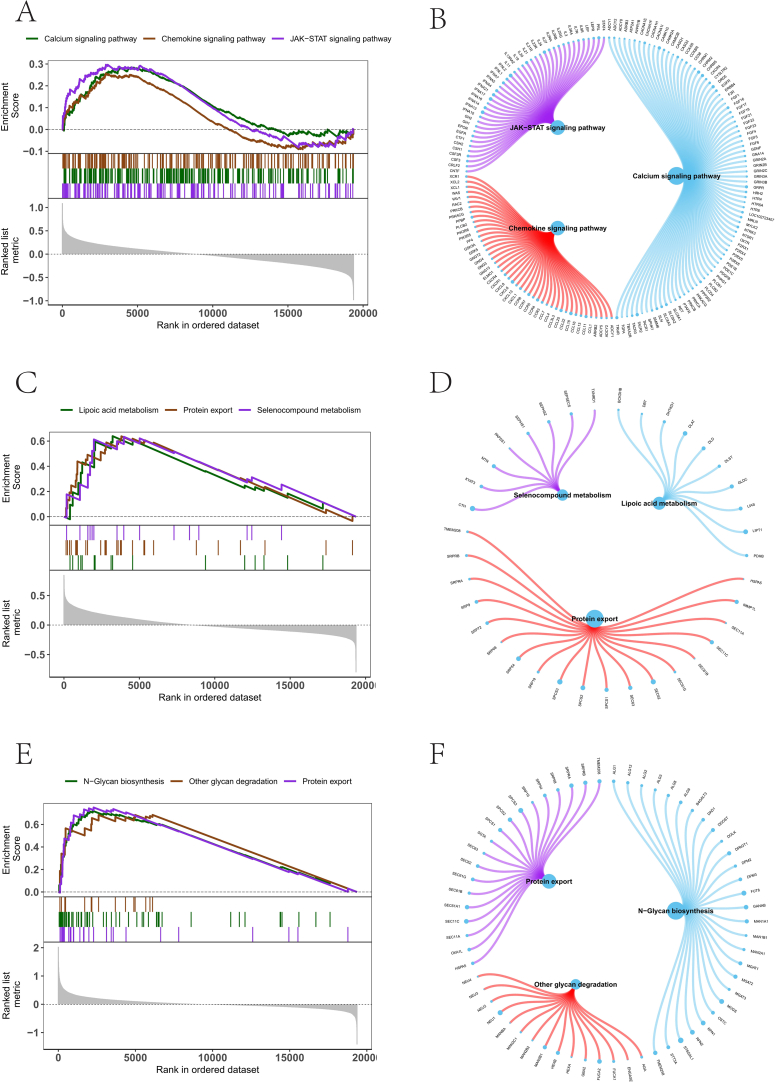


**Fig. S7:**
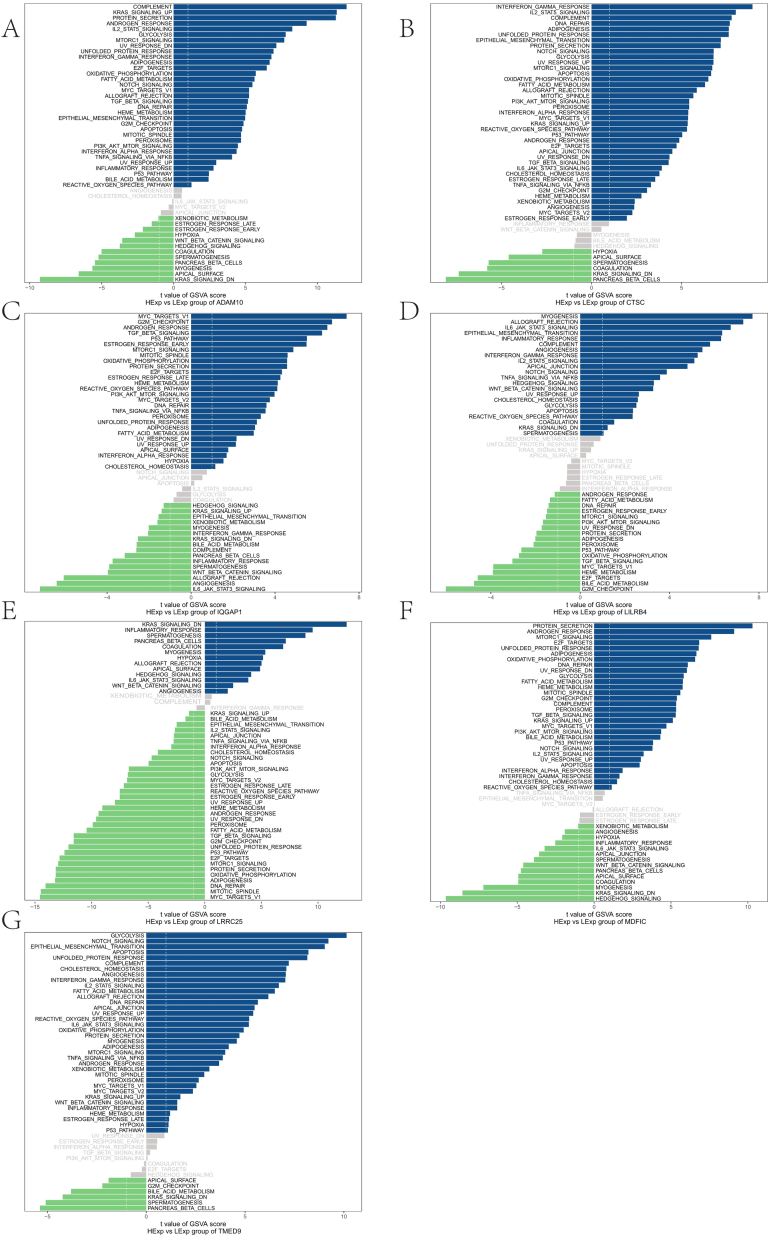


**Fig. S8:**
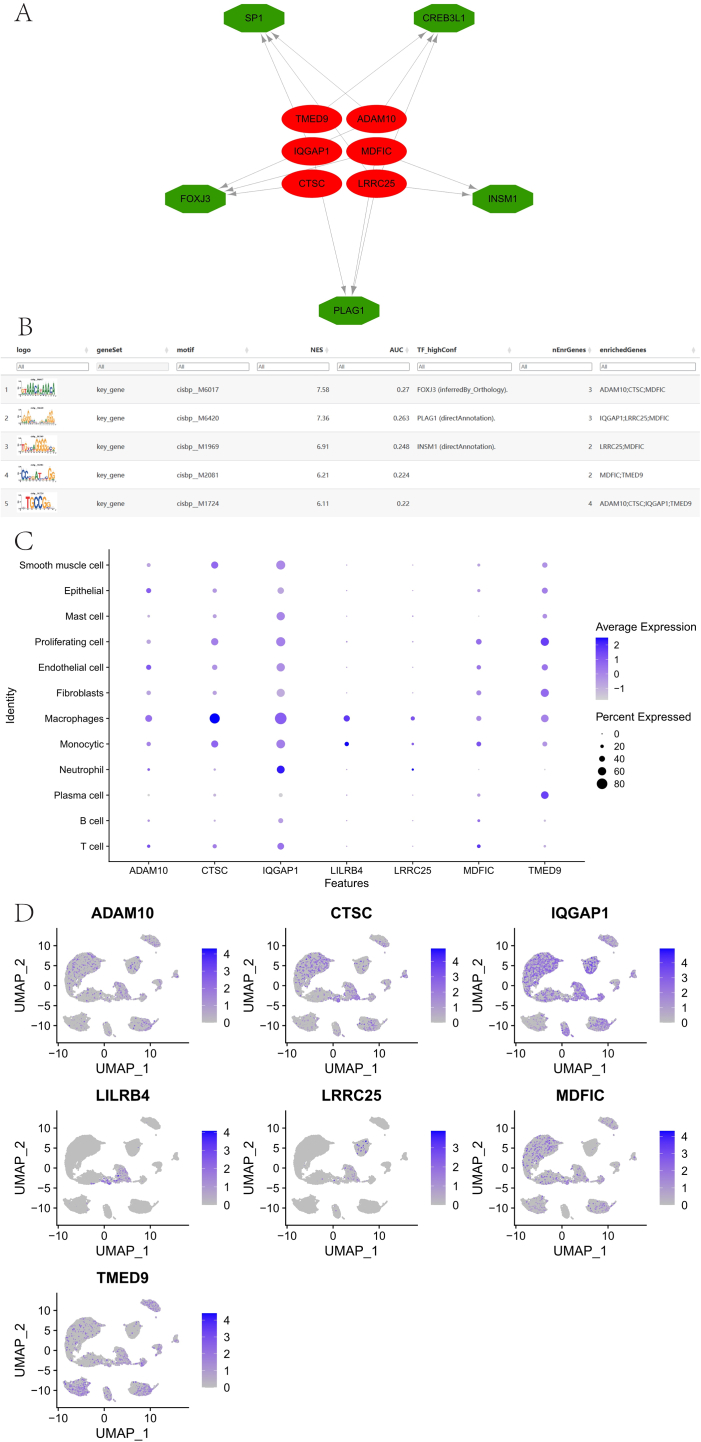


**Fig. S9:**