# Supplementary material for: Single-cell transcriptomics unveils iron dysregulation in macrophages: Implicated genes in periodontitis pathogenesis identified via Mendelian randomization
Source: Genes Dis. 2025 Jan 22;13(1):101544. doi: 10.1016/j.gendis.2025.101544 (PMC12466136; doi:10.1016/j.gendis.2025.101544)
Supplement: Multimedia component 1 [file mmc1.docx]

**Important Materials and Methods**

**Data acquisition**

**Data Acquisition from the GEO Repository**
The Gene Expression Omnibus (GEO), a publicly available repository maintained by the National Center for Biotechnology Information (NCBI), archives a wealth of gene expression data. For this study, the Series Matrix File related to dataset GSE16134 was extracted from the GEO database using the annotation file GPL570. The dataset includes the gene expression profiles of 310 individuals, comprising 69 controls and 241 disease-affected subjects. Additionally, the single-cell data file, identified as GSE171213 and originating from the same patient cohort, was retrieved. This dataset contains single-cell expression profiles for 9 individuals, divided equally between 4 controls and 5 disease-affected subjects.

**Source of eQTL Data**The expression quantitative trait loci (eQTL) data used in this study were obtained from the publicly available eQTLGen consortium database (<https://www.eqtlgen.org>). This consortium focuses on identifying the genetic determinants of gene expression in blood, aiming to uncover the genetic basis of complex traits. The current phase of the consortium’s project is centered on conducting extensive genome-wide meta-analyses of blood-derived samples.**^1^**

**Research Findings and Genome-Wide Association Studies (GWAS)**This study’s GWAS analysis predominantly involved individuals of European ancestry. Data were obtained from the Finnish FinnGen project database, specifically the FinnGen PERIODON CHRON dataset. FinnGen is a major genetic research initiative aimed at analyzing genetic and health-related data within European populations. GWAS data related to periodontitis were acquired from FinnGen, consisting of 3,046 disease cases and 195,395 controls.

**Single cell sequencing analysis**

The expression profile was first analyzed using the Seurat package, with low-expression genes filtered out. Sequential standardization, normalization, PCA, and UMAP analyses were performed to reduce dimensionality and visualize clusters. Cellular clusters were classified and annotated using the Seurat toolkit, with a focus on highlighting cells pivotal to disease progression. Distinctive genes specific to each cell subtype were subsequently identified from single-cell RNA sequencing profiles by configuring the 'FindAllMarkers' function with a log fold change threshold of 0.25.

**Functional Analysis of GO and KEGG**
Functional characterization of differentially expressed genes was performed using the 'ClusterProfiler' library in R to investigate their functional relationships. The Gene Ontology (GO) and Kyoto Encyclopedia of Genes and Genomes (KEGG) databases were used to explore relevant functional domains.**^2^** Pathways with p-values and q-values below 0.05 were considered statistically significant in both GO and KEGG analyses.

**Mendelian Randomization Analysis**
The FinnGen repository, accessible at [*https://www.finngen.fi/fi*](https://www.finngen.fi/fi), offers a rich compilation of aggregated statistics derived from a multitude of Genome-Wide Association Studies (GWAS). Within this database, SNPs with a significance level of P<1e^-8^ linked to periodontitis were cross-referenced with those that have a similar significance level and are implicated in modulating key genes, as identified through single-cell sequencing. This process involved retaining SNPs with a linkage disequilibrium R2 value below 0.001 within a clumping window of 10,000kb and a secondary p-value threshold of p2<5^e-8^, to qualify as instrumental variables (IV). Subsequently, these SNPs underwent inverse variance weighting (IVW), which combines meta-analysis with the Wald approximation for each SNP, MR Egger analysis, which presupposes that the strength of the instrument is unrelated to the direct effects (InSIDE), weighted median analysis, which is capable of correctly estimating causality in scenarios where up to 50% of IVs are not valid, and weighted mode analysis, recognized for its enhanced capability to sense causal impacts with reduced prejudice and a lesser Type I error rate compared to MR-Egger regression. A singular statistical approach was applied to assess the robustness of the causal relationship when only one method was available for a SNP, utilizing the Wald ratio. This provided a consolidated measure of the influence that all cis-acting and some distal gene expression have on periodontitis in whole blood. Ultimately, the causal relationships identified through this process were subjected to verification and further analysis using the leave-one-out cross-validation technique.**^3^**

**Sensitivity Analysis and Heterogeneity Testing**
The robustness of causal relationships was evaluated using leave-one-out sensitivity analysis, which excluded individual SNPs sequentially and recalculated the total effect of the remaining markers. This approach provided new point estimates and confidence intervals, allowing the assessment of each SNP’s impact on overall results. A Q statistic, derived from the weighted sum of squared result sizes and standard errors, was used to test heterogeneity. Q values >0.05 indicated no significant heterogeneity, suggesting consistent effects of the SNPs on disease risk.

**Immune Cell Infiltration Analysis**The CIBERSORT technique, based on support vector regression, was employed to estimate immune cell abundance in the samples through a deconvolution approach. This method utilizes expression profiles of immune cell markers, encompassing 547 genes, to identify 22 subpopulations of human immune cells, including T cells, B cells, plasma cells, and various myeloid groups. In this study, CIBERSORT was used to analyze the patient data, estimating the relative abundance of immune cell subsets. Additionally, Pearson correlation analysis was conducted to assess the relationships between gene expression levels and immune cell infiltration patterns.**^4^**

**GSEA Pathway Enrichment Analysis**Gene Set Enrichment Analysis (GSEA) was performed to examine alterations in signaling pathways between groups with high and low expression of specific genes. The analysis utilized the Molecular Signatures Database (MSigDB),**^5^** version 7.0, as the reference gene set. GSEA enabled comparative analysis of differential gene expression across subtypes, revealing significantly enriched gene sets (adjusted p-value < 0.05). This approach is widely employed for integrating disease classification with insights into biological processes.

**GSVA (Gene Set Variation Analysis)**Gene Set Variation Analysis (GSVA) was conducted to investigate pathway-level differences between groups exhibiting elevated versus reduced gene expression. The benchmark gene set, derived from MSigDB (version 7.0), provided a framework for assessing subtype-specific pathways. This analysis identified pathways with significant enrichment (adjusted p-value < 0.05), allowing the differentiation of molecular processes associated with gene expression variations. GSVA is particularly suited for studies linking disease subtypes to biological mechanisms.

**Transcriptional Regulation Analysis of Key Genes**The transcriptional regulation of key genes was explored using the 'RcisTarget' package in R, which predicts transcription factor activity through DNA motif analysis. The normalized enrichment score (NES) for each motif was calculated based on its occurrence in gene sets. To expand the scope, additional annotations were inferred from motif similarity and genetic sequences. The influence of each motif was quantified using recovery curve analysis, comparing gene sets with ranked motifs. NES values were derived from the area under the curve (AUC) distribution across all motifs, providing insights into the regulatory networks governing key genes.**^6^**

**Statistical Analysis**Robust Mendelian randomization (MR) analysis relied on three key assumptions: (1) the correlation assumption, requiring the instrumental variable to be strongly associated with the exposure; (2) the independence assumption, ensuring the instrumental variable is unrelated to confounding factors; and (3) the exclusivity assumption, stipulating that the variable influences the outcome only through the exposure. Statistical evaluations were conducted using R version 4.2.2, employing a two-tailed approach. Results with p-values < 0.05 were considered statistically significant.

**Results:**

**Single cell sequencing analysis reveals that macrophage cells are most impacted by iron dysregulation**

First, the data samples were filtered using nFeature_RNA and nCount_RNA (nFeature_RNA > 200 & nFeature_RNA < 4000, percent.mt < 40, and nCount_RNA < 20000), following the reading of the expression profile using the Seurat software. A total of 32,785 cells (Fig. S1A-B) were obtained after filtering, which displayed 10 genes with the highest standard deviation (Fig. S1C). The data were standardized, followed by PCA and harmonization (Fig. S1D-F). The spatial relationships among 23 cell clusters were determined using UMAP analysis (Fig. 1A). Each subtype was further annotated with T cells, B cells, plasma cells, neutrophils, monocytes, macrophages, fibroblasts, endothelial cells, proliferation cells, mast cells, epithelial cells, and smooth muscle cells (Fig. 1B). Classic markers of 12 types of cells (Fig. 1C) and cell proportions corresponding to groups (Fig. 1D) are presented.

A total of 514 genes related to iron metabolism were identified from the literature to evaluate the iron metabolism score at the single-cell level using ssGSEA. Macrophages exhibited the most significant difference in iron metabolism scores between periodontitis groups and controls. We then performed differential gene expression (DGE) analysis between the case and control groups of macrophages (adjusted P < 0.05). Ultimately, 419 related differentially expressed genes were retained for further analysis (Fig. 1E).

**Differentially expressed genes in macrophage are enriched in immune system processes**

An in-depth investigation of the differentially expressed genes was performed to explore their involvement in biological pathways. The Gene Ontology (GO) enrichment analysis showed significant enrichment of these genes in pathways associated with the negative regulation of immune system processes, lysosome organization, and receptor-mediated endocytosis (Fig. S2A). Furthermore, the Kyoto Encyclopedia of Genes and Genomes (KEGG) enrichment analysis demonstrated notable concentration of the differentially expressed genes in pathways including Endocytosis, Phagosome, and Lysosome (Fig. S2B).

**Mendelian randomization analysis shows that seven genes are implicated in the cause of periodontitis**

To identify pivotal genes influencing periodontitis, we focused on differentially expressed genes within macrophages and identified Single Nucleotide Polymorphisms (SNPs) significantly linked to these genes with a stringent threshold of (P<1e-8). These associations were corroborated across two databases: the periodontitis GWAS study within the Finngen repository, comprising 3,046 controls and 195,395 cases, and the eQTL blood database. SNPs present in both databases with a linkage disequilibrium R2 of less than 0.001 and a secondary p-value threshold of (p2<5e-8) were subjected to further analysis, yielding 371 gene-outcome causal pairs as illustrated in. Subsequent filtering through Mendelian randomization analysis pinpointed causal relationships for seven gene pairs that corresponded with positive eQTL outcomes, depicted in with an Inverse Variance Weighted (IVW) p-value of less than (0.05). The seven genes in question are ADAM10, CTSC, IQGAP1, LILRB4, LRRC25, MDFIC, and TMED9. Specifically, ADAM10(1.152; 1.015−1.306;p=0.028), IQGAP1(1.119; 1.015−1.234;p=0.024), LILRB4(1.126; 1.006−1.260;p=0.040), and TMED9(1.143; 1.004−1.302; p= 0.044) are correlated with an elevated risk of periodontitis, whereas CTSC(0.893; 0.818−0.976;p=0.012), LRRC25(0.914; 0.837−0.999;p=0.046), and MDFIC(0.714; 0.550−0.928;p=0.012) are indicative of a decreased risk. (Fig 1F) A sensitivity analysis was conducted on these seven gene-causal relationships using the leave-one-out method to ascertain their reliability, with results indicating that the exclusion of any single SNP did not significantly alter the overall confidence intervals, thereby confirming the robustness of these relationships as shown in (Fig S3). Heterogeneity tests were then utilized to evaluate the consistency of the effects across the seven gene pairsA Q value was obtained by summing the weighted squares of the effect sizes and standard errors for every SNP.. The Q values for all gene pairs exceeded 0.05, suggesting that their impacts on disease susceptibility are statistically homogeneous. Consequently, these seven genes have been identified as key candidates for our subsequent research endeavors.

**Key genes implicated in the cause of periodontitis are closely related to immune cell functions that are implicated in immune infiltration and microenvironment**

The microenvironment, a multifaceted environment made up of immune cells, extracellular matrix elements, various growth factors, inflammatory mediators, and special physicochemical characteristics, has a significant impact on the identification of diseases, the prognosis of patients, and the responsiveness of clinical interventions.. Through an examination of the interactions between key genes and immune cell infiltration within the periodontitis dataset, coupled with an exploration of the underlying molecular mechanisms influencing the progression of periodontitis, we have elucidated the distribution of immune cells among patients and their interrelationships, as depicted in the correlation analysis ((Fig 1G-H). The study findings reveal substantial disparities in differential gene expression across various groups, including activated and resting Dendritic cells, Macrophages M0 and M1, Mast cells, Monocytes, Neutrophils, NK cells, Plasma cells, and subsets of T cells such as CD8, follicular helper, gamma delta, and regulatory T cells (Tregs) (Fig 1I).Our subsequent investigation into the associations between key genes and specific immune cells yielded several significant correlations. For instance, ADAM10 displayed a notable positive association with T cells CD4 memory resting, Neutrophils, and T cells gamma delta, while showing a negative correlation with T cells CD8, Tregs, and Monocytes. CTSC was found to have a positive correlation with Neutrophils, T cells gamma delta, and Macrophages M1, and an inverse relationship with T cells CD8 and Tregs. IQGAP1 was positively linked with resting Dendritic cells, Mast cells, and Macrophages M1,

and showed both positive and negative correlations with Plasma cells, Macrophages M2, and resting NK cells. LILRB4 exhibited a positive correlation with Macrophages M0, Plasma cells, and resting NK cells, as well as with Dendritic cells in both resting and activated states. LRRC25 demonstrated a negative correlation with activated T cells CD4 memory, resting NK cells, and CD8 T cells, and also with resting Mast cells, follicular helper T cells, and Dendritic cells. MDFIC and TMED9 were found to be positively correlated with T cells CD4 memory resting, T cells gamma delta, B cells ©, and negatively with T cells CD8, Macrophages M0, and Tregs, as illustrated in (Fig 1J).

Concurrently, leveraging data from the TISIDB database, we established the correlation between key genes and an array of immune factors, encompassing immunosuppressive and immunostimulatory agents, chemokines, and receptors. These analyses underscore the pivotal role that key genes play in relation to immune cell infiltration levels and their consequent impact within the immune microenvironment (Fig S4).

**GSEA pathway enrichment analysis shows key genes are enriched in important immune pathways**

Then, in order to determine the molecular processes via which these seven crucial genes may impact the development of periodontitis, we further explored the distinct signaling pathways that are abundant within them. The Gene Set Enrichment Analysis (GSEA) findings indicated that ADAM10 is implicated in pathways such as the IL-17 signaling pathway, NF-kappa B signaling pathway, and TNF signaling pathway, among others, as illustrated in (Fig S5A-B); CTSC was found to be associated with pathways including Other glycan degradation, Proteasome, and Protein export, as depicted in (Fig S5C-D); IQGAP1 was linked to pathways such as the B cell receptor signaling pathway, IL-17 signaling pathway, and NF-kappa B signaling pathway, as shown in (Fig S5E-F); LILRB4 was observed to be enriched in similar pathways to IQGAP1, including the B cell receptor signaling pathway, IL-17 signaling pathway, and NF-kappa B signaling pathway, represented in (Fig S5G-H); LRRC25 was identified in pathways like the Calcium signaling pathway, Chemokine signaling pathway, and JAK−STAT signaling pathway, among others, as detailed in (Fig S6A-B); MDFIC was shown to be involved in pathways such as Lipoic acid metabolism, Protein export, and Seleno compound metabolism, as demonstrated in (Fig S6C-D); TMED9 was found to be enriched in pathways including N-Glycan biosynthesis, Other glycan degradation, and Protein export, as indicated in (Fig S6E-F).

**GSVA Pathway Enrichment Analysis of Key Genes**

The GSVA outcomes have shed light on the pathways significantly associated with ADAM10, which comprise the COMPLEMENT pathway, KRAS_SIGNALING_UP, PROTEIN_SECRETION, and additional pathways as illustrated in (Fig S7A). For CTSC, the pathways include the INTERFERON_GAMMA_RESPONSE, IL2_STAT5_SIGNALING, COMPLEMENT, and more, as represented in (Fig S7B). IQGAP1 is linked to pathways such as MYC_TARGETS_V1, G2M_CHECKPOINT, ANDROGEN_RESPONSE, and other pathways, depicted in (Fig S7C). LILRB4 is found to enrich pathways like MYOGENESIS, ALLOGRAFT_REJECTION, IL6_JAK_STAT3_SIGNALING, and others, shown in (Fig S7D). LRRC25 shows enrichment in pathways including MYOGENESIS, ALLOGRAFT_REJECTION,

IL6_JAK_STAT3_SIGNALING, and additional pathways, as indicated in (Fig S7E). MDFIC is associated with pathways such as PROTEIN_SECRETION, ANDROGEN_RESPONSE, MTORC1_SIGNALING, and further pathways, detailed in (Fig S7F). Lastly, TMED9 is linked to pathways like GLYCOLYSIS, NOTCH_SIGNALING, EPITHELIAL_MESENCHYMAL_TRANSITION, and other pathways, as demonstrated in (Fig S7G).

**Transcriptional regulation analysis of key genes**

Using the seven key genes as our collection of genes for this study, we found that they are regulated by common regulatory pathways, such as the action of different transcription factors. Thus, these transcription factors were the subject of an enrichment study employing cumulative recovery curves. Based on the analysis of motif-TF annotation and the identification of important genes, cisbp__M6017 is associated with the motif that has the highest normalized enrichment score (NES: 7.58). We display every enriched motif for each of the important genes in (Fig S8A-B) together with the corresponding transcription factors.

**Expression profile of key genes in single cell data**

We analyzed the expression of key genes in single cells and showed that key genes are expressed in Smooth muscle cell, Epithelial, Mast cell, Proliferating cell, Endothelial cell, Fibroblasts, Macrophages, Monocytic, Neutrophil, Plasma cell, B cell, T cell (Fig S8C-D).

**Additional Methods and Results**

**GEO Dataset Analysis:**

To deepen our understanding of the interplay between macrophage gene expression and iron metabolism in the context of periodontitis, we leveraged the GSE24897 dataset from the Gene Expression Omnibus (GEO) repository. This dataset encompasses gene expression profiles of human macrophages exposed to P. gingivalis, a bacterium central to periodontal disease pathology. We downloaded the raw CEL files and processed them using the Robust Multichip Average (RMA) algorithm for background correction, normalization, and summarization. Subsequently, we employed the limma package in R, a widely-used tool for the analysis of gene expression microarray data, to perform differential gene expression analysis. Identifying genes with adjusted p-values < 0.05 as significantly differentially expressed. To further investigate the biological relevance of these differentially expressed genes, we conducted Gene Set Enrichment Analysis (GSEA) and Gene Ontology (GO) enrichment analysis using the clusterProfiler R package. These analyses aimed to uncover pathways and biological processes related to iron metabolism that were significantly enriched among the differentially expressed genes. (Fig S9A-C).

**STRING Database for Protein-Protein Interactions:**

To explore the functional associations and potential regulatory networks involving the seven key genes associated with periodontitis, we utilized the STRING database. This database is a valuable resource for known and predicted protein-protein interactions, providing a comprehensive view of molecular associations. We entered our genes of interest into STRING and obtained a network of interactions with a minimum required interaction confidence score of 0.7, which STRING defines as high confidence. This score indicates a strong likelihood that the interactions are true, based on experimental validation, database records, or co-expression evidence.The resulting PPI network was directly visualized within the STRING interface, which presents a clear and intuitive graphical representation of the interactions. To gain further insights into the biological significance of these interactions, we conducted a functional enrichment analysis specifically on the proteins involved in the network. We focused on the enrichment analysis results for our seven key genes and their connected proteins. This analysis helped to elucidate the potential roles of these genes in the periodontitis. (Fig S9D)

**GeneCards Database Utilization:**

GeneCards, a database that amalgamates a wealth of information on human genes, their products, and their roles in diseases, was instrumental in our quest to gather comprehensive data on genes pertinent to iron homeostasis. We conducted a targeted search within GeneCards for genes annotated with terms related to iron metabolism, yielding a curated list that was cross-referenced with our seven key genes identified in the study. This cross-referencing was performed to validate and augment our findings with existing knowledge on iron metabolism, thereby reinforcing the biological significance of our gene selection. The data extracted from GeneCards included gene summaries, protein information, and gene expression profiles, which collectively contributed to a more nuanced understanding of the genes' roles in the iron metabolism. (Fig S9E)

**References**

1. Grundberg E, Small KS, Hedman ÅK, et al. Mapping cis- and trans-regulatory effects across multiple tissues in twins. Nat Genet. 2012;44(10):1084-1089.
2. Ashburner M, Ball CA, Blake JA, et al. Gene ontology: tool for the unification of biology. The Gene Ontology Consortium. Nat Genet. 2000;25(1):25-29.
3. Zhu J, Zhou D, Nie Y, et al. Assessment of the bidirectional causal association between frailty and depression: A Mendelian randomization study. J Cachexia Sarcopenia Muscle. 2023;14(5):2327-2334.
4. Newman AM, Liu CL, Green MR, et al. Robust enumeration of cell subsets from tissue expression profiles. Nat Methods. 2015;12(5):453-457.
5. Subramanian A, Tamayo P, Mootha VK, et al. Gene set enrichment analysis: a knowledge-based approach for interpreting genome-wide expression profiles. Proc Natl Acad Sci U S A. 2005;102(43):15545-15550.
6. Luscombe NM, Babu MM, Yu H, Snyder M, Teichmann SA, Gerstein M. Genomic analysis of regulatory network dynamics reveals large topological changes. Nature. 2004;431(7006):308-312.


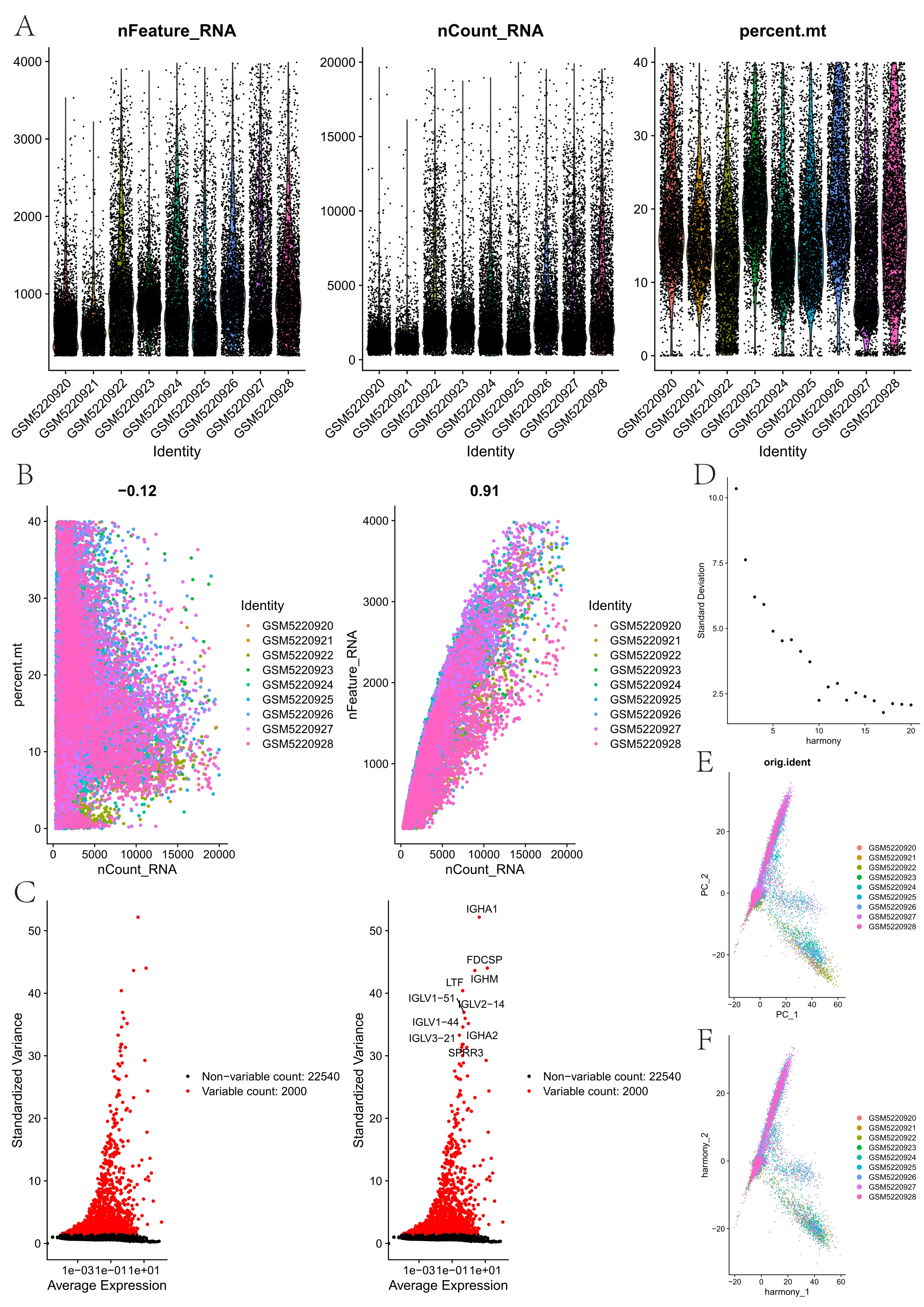


**Figure S1:** Single-cell pre-processing. **(A)** Single-cell quality control, displaying the number of cells, genes, and sequencing depth for each sample. **(B)** The left panel shows the relationship between cell sequencing depth and mitochondrial content, while the right panel illustrates the positive correlation between sequencing depth and gene count. **(C)** We identified genes with significant intercellular variability and constructed a feature variance plot. **(D)** Variance ranking plot for each principal component (PC). **(E, F)** Presentation of principal component analysis (PCA) and the distribution of PCs, where dots represent cells and colors represent samples.


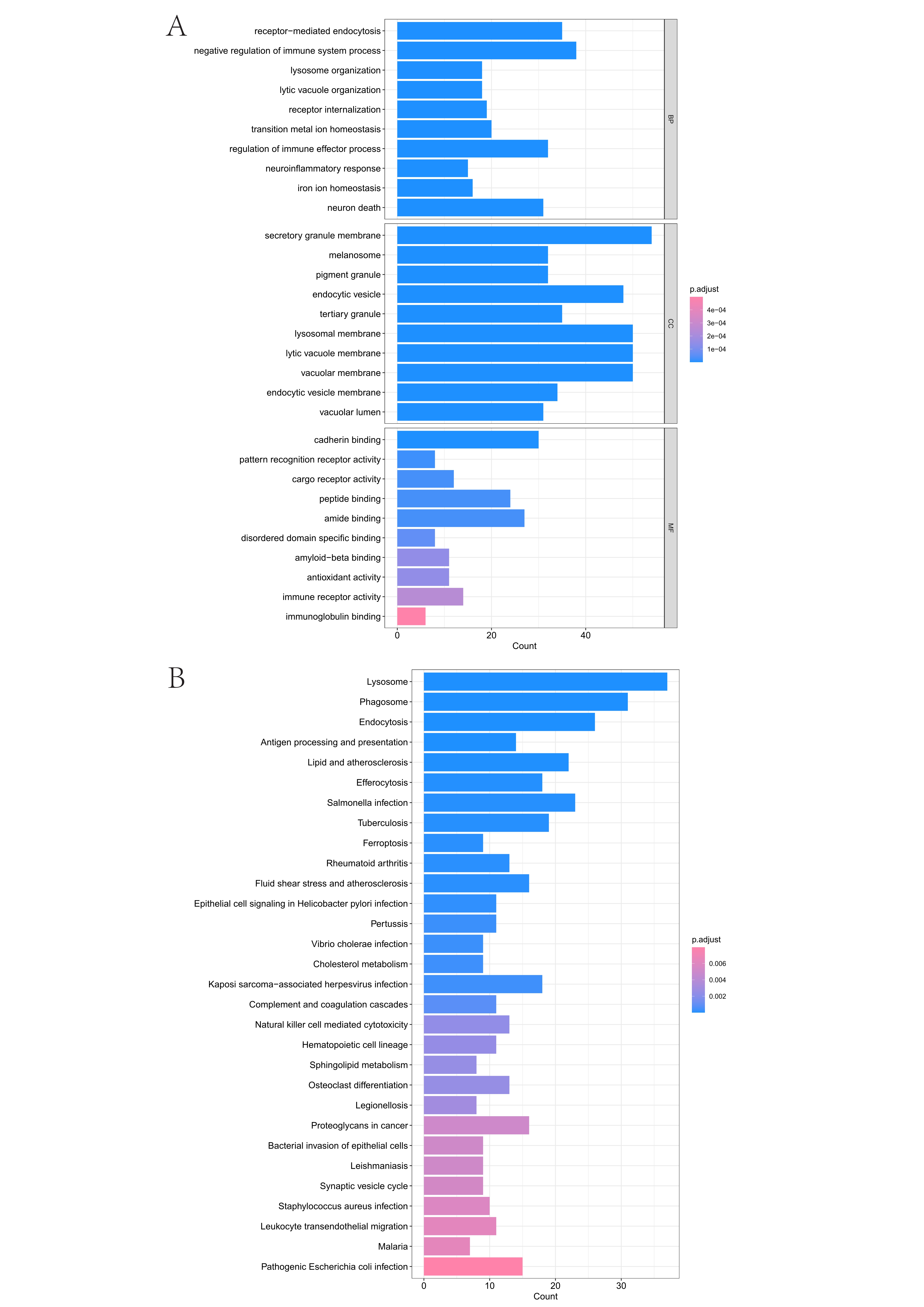


**Figure S2:** Enrichment Analysis. **(A, B)** GO (Gene Ontology) and KEGG (Kyoto Encyclopedia of Genes and Genomes) enrichment analysis based on the ClusterProfiler.


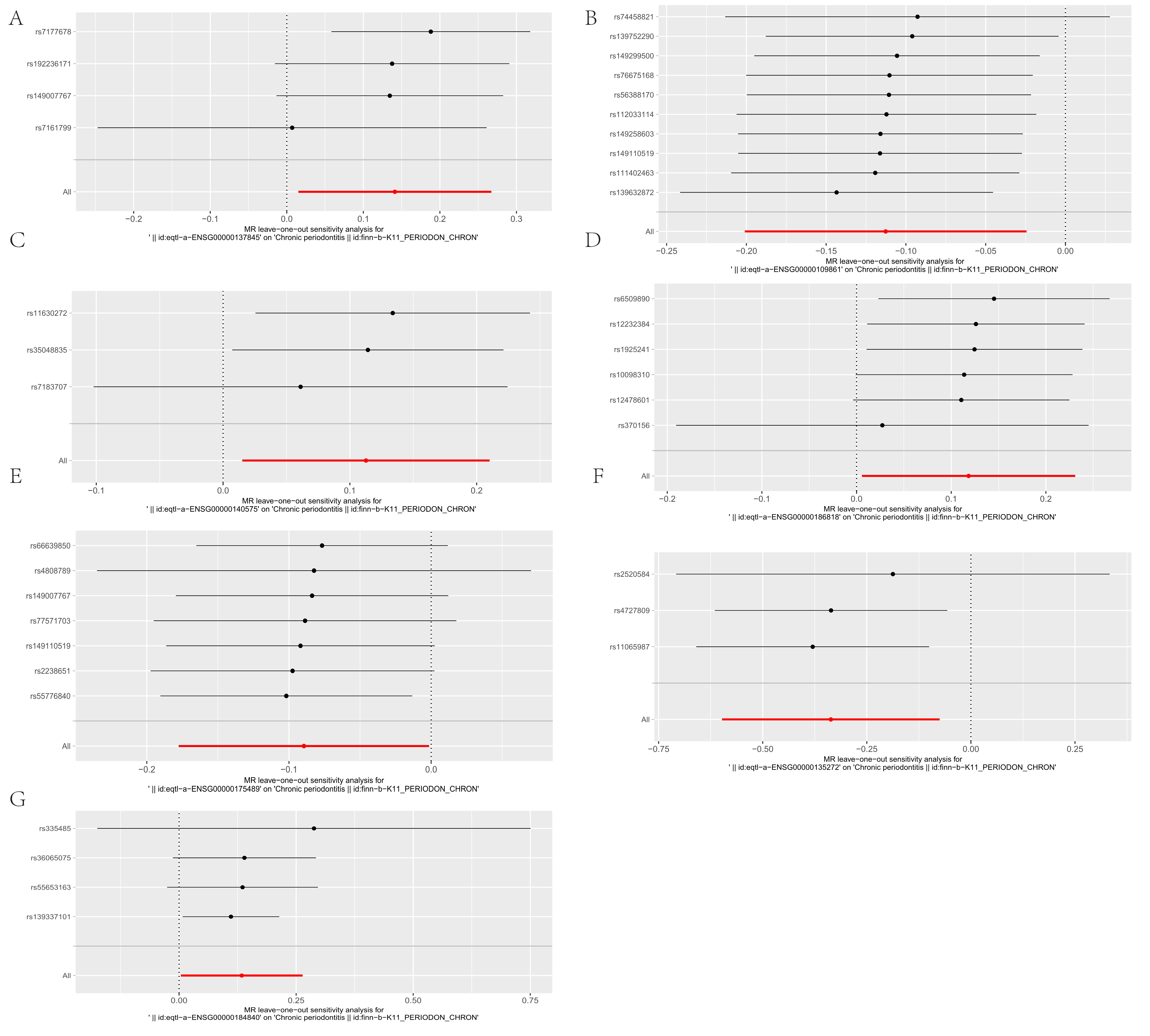


**Figure S3:** Leave-out Test. **(A-G)** Forest plots of the leave-out test for the single nucleotide polymorphisms (SNPs) corresponding to key genes.


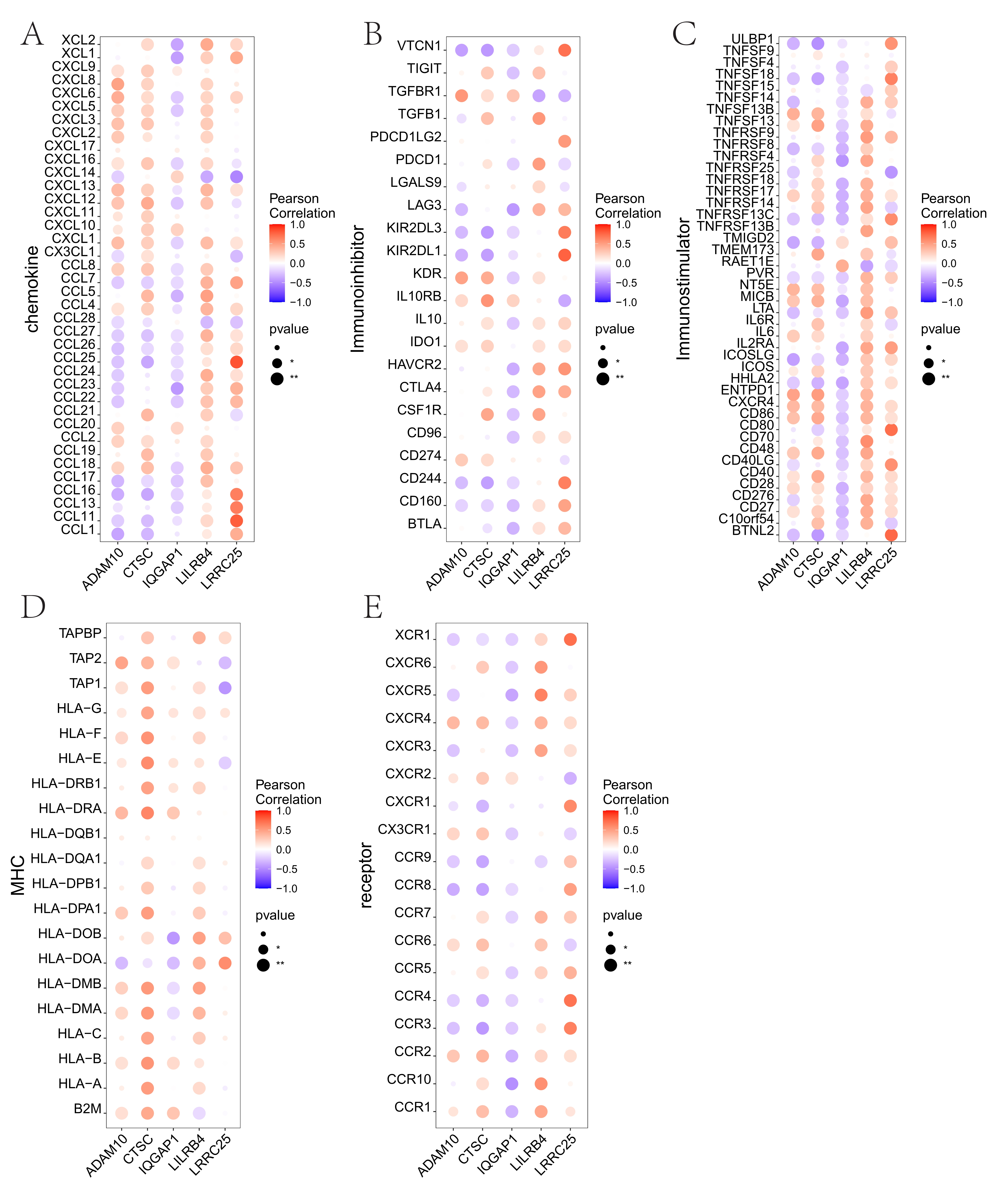


**Figure S4:** Relationship between Key Genes and Immune Factors. **(A-E)** Correlation of key genes with chemokines, immunoinhibitors, immunostimulators, MHC (major histocompatibility complex), and receptors.


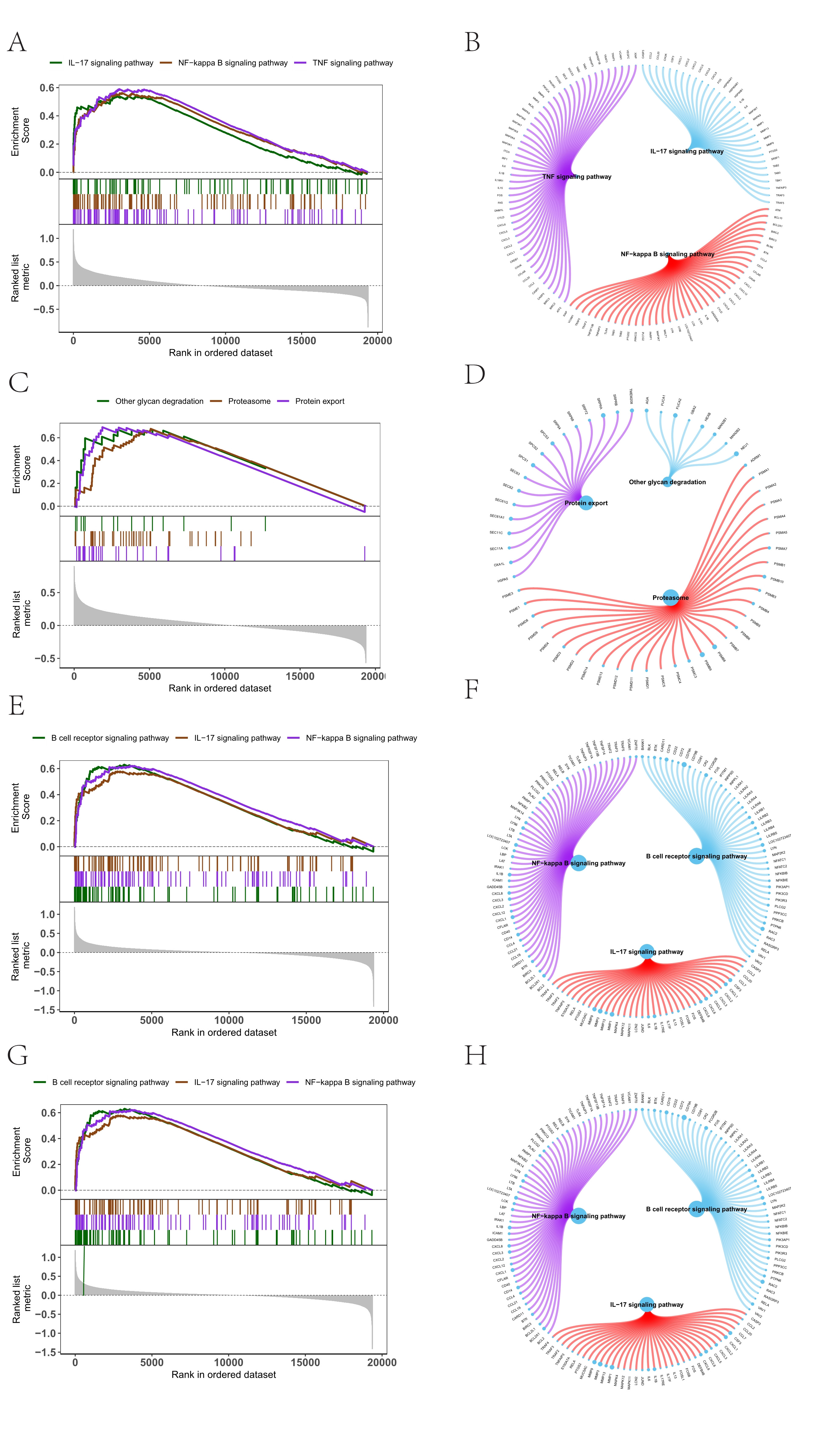


**Figure S5:** Gene Set Enrichment Analysis (GSEA) of Key Genes. **(A-H)** KEGG signaling pathways associated with key genes, as well as the regulation of these pathways and the genes involved.


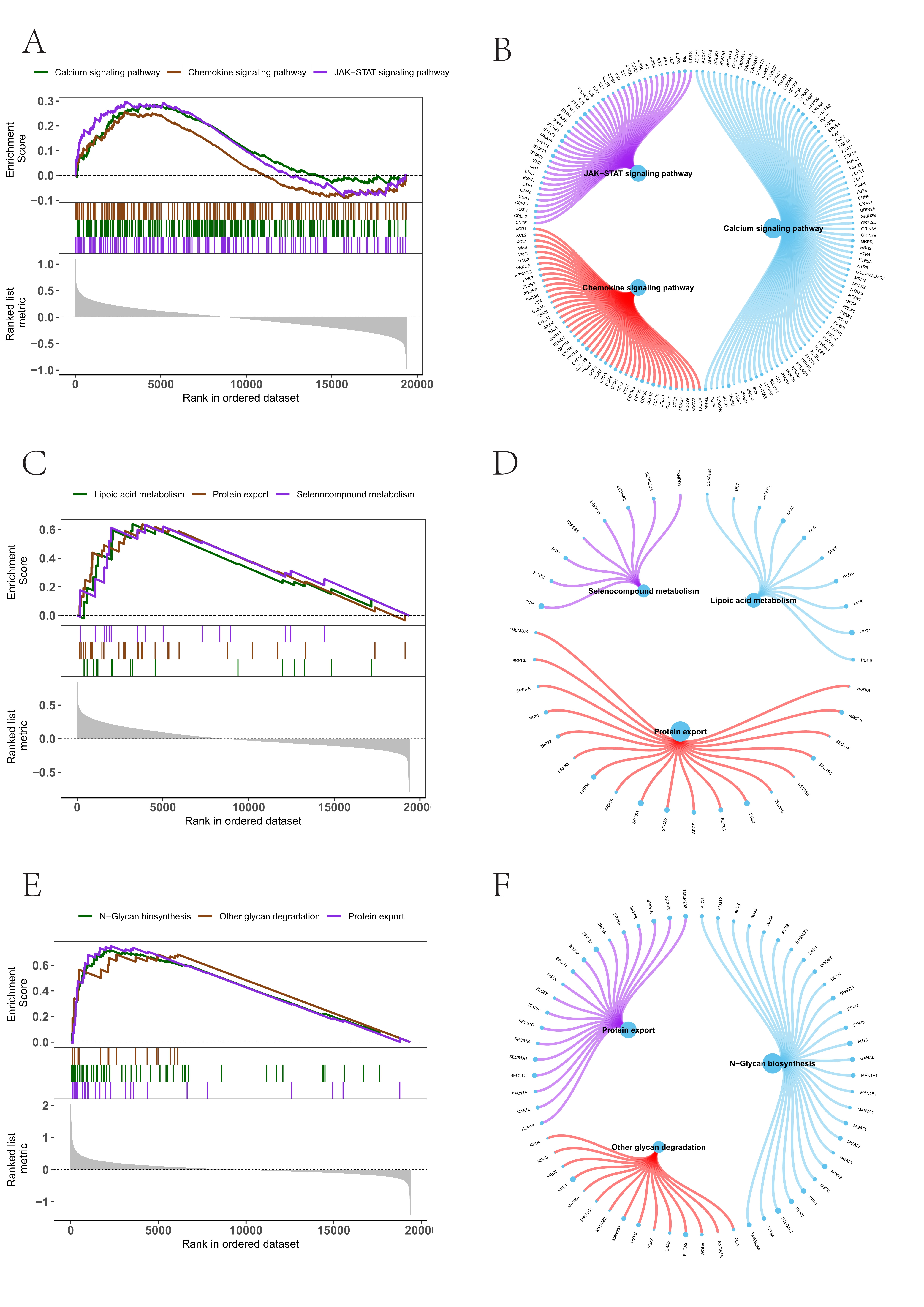


**Figure S6:** Gene Set Enrichment Analysis (GSEA) of Key Genes. **(A-F)** KEGG signaling pathways implicated by key genes, along with the regulation of these pathways and the genes involved.


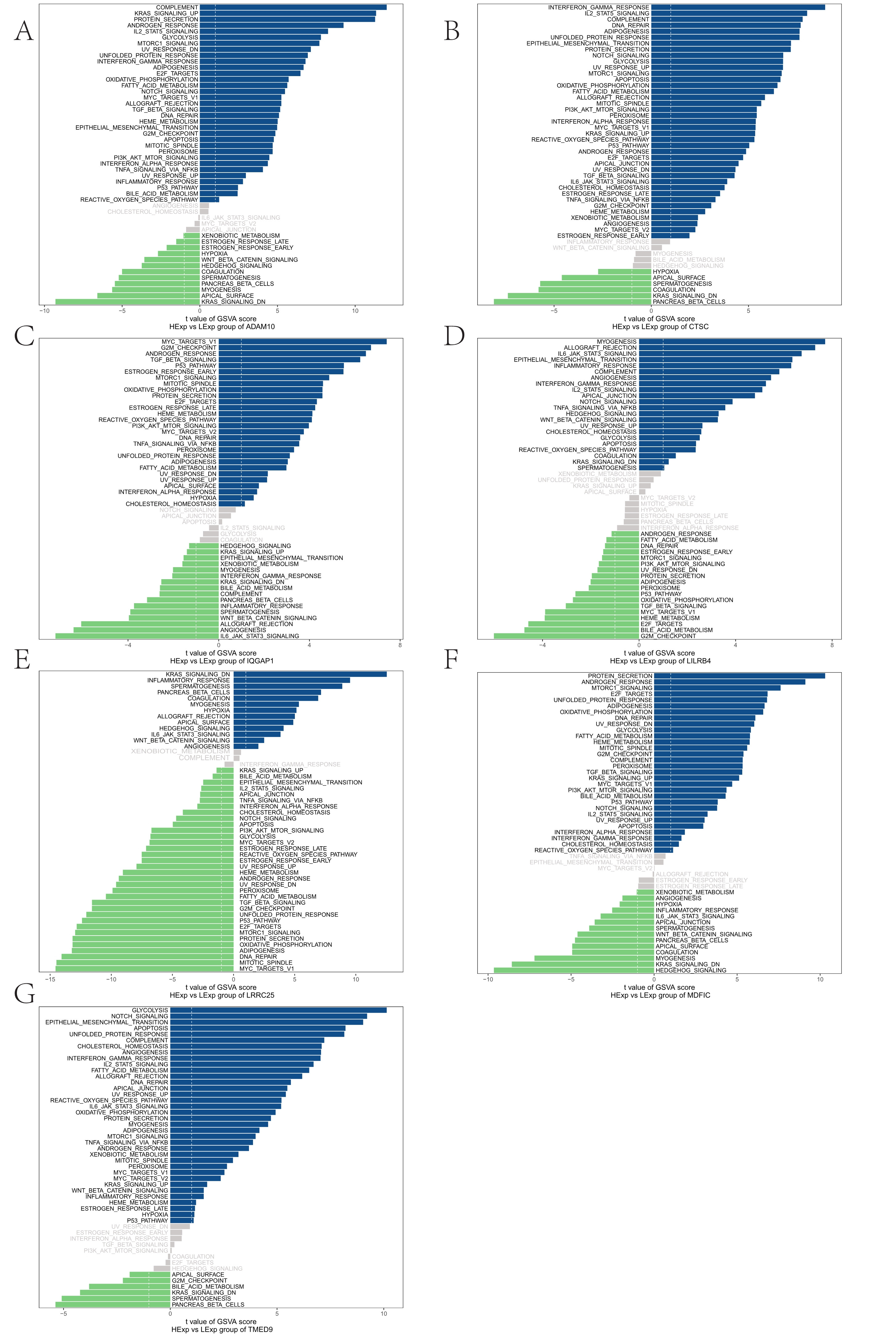


**Figure S7:** Gene Set Variation Analysis (GSVA) of Key Genes. **(A-G)** GSVA analysis of key genes, with blue representing the high expression of genes involved in signaling pathways and green indicating the low expression of genes involved, using the hallmark gene set as the background.


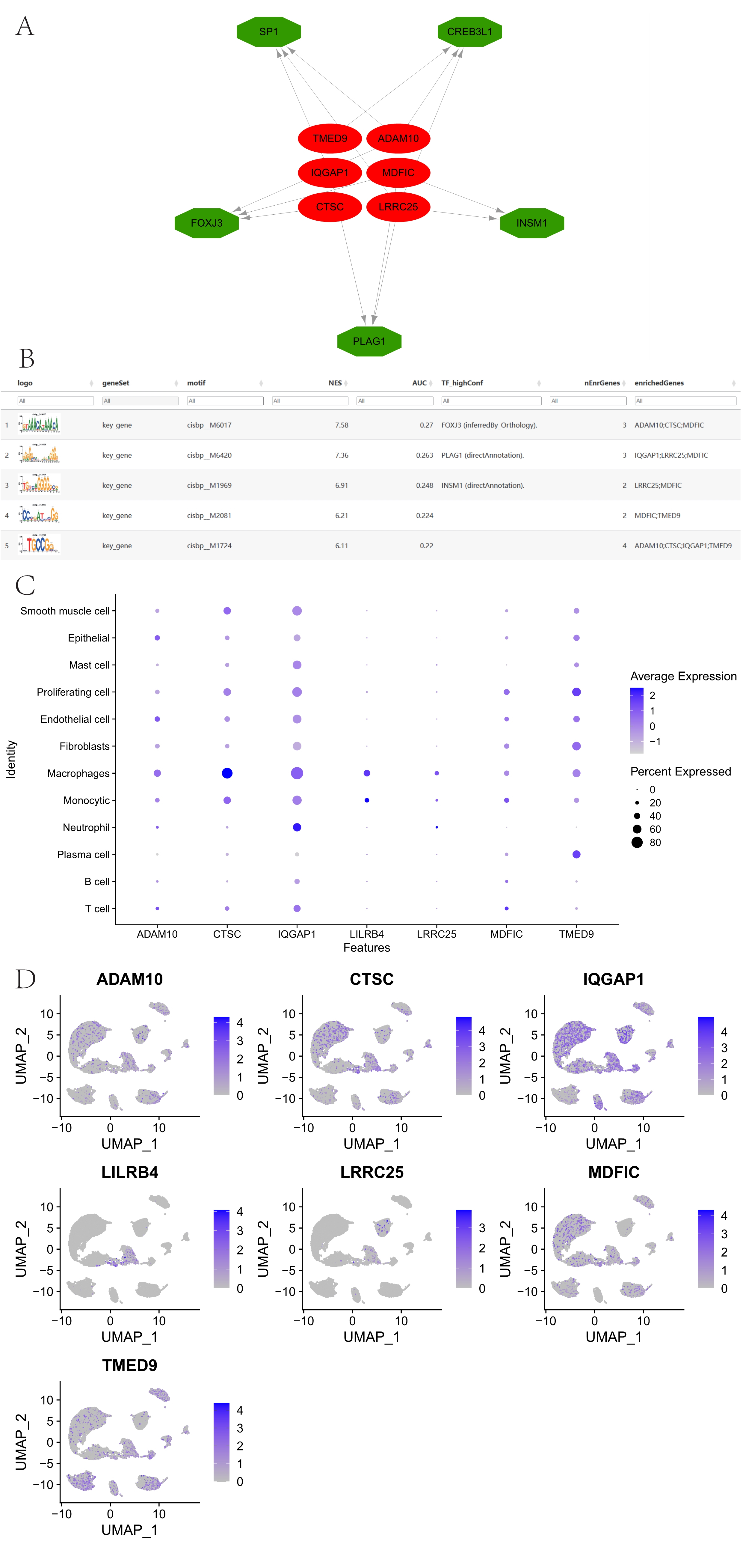


**Figure S8:** Transcriptional Regulatory Network and Single-Cell Expression of Key Genes. **(A)** The transcriptional regulatory network of key genes, with red representing key genes and green representing transcription factors. **(B)** A display of all enriched motifs and their corresponding transcription factors associated with key genes. **(C, D)** An overview of the expression of key genes in single-cell contexts.

**
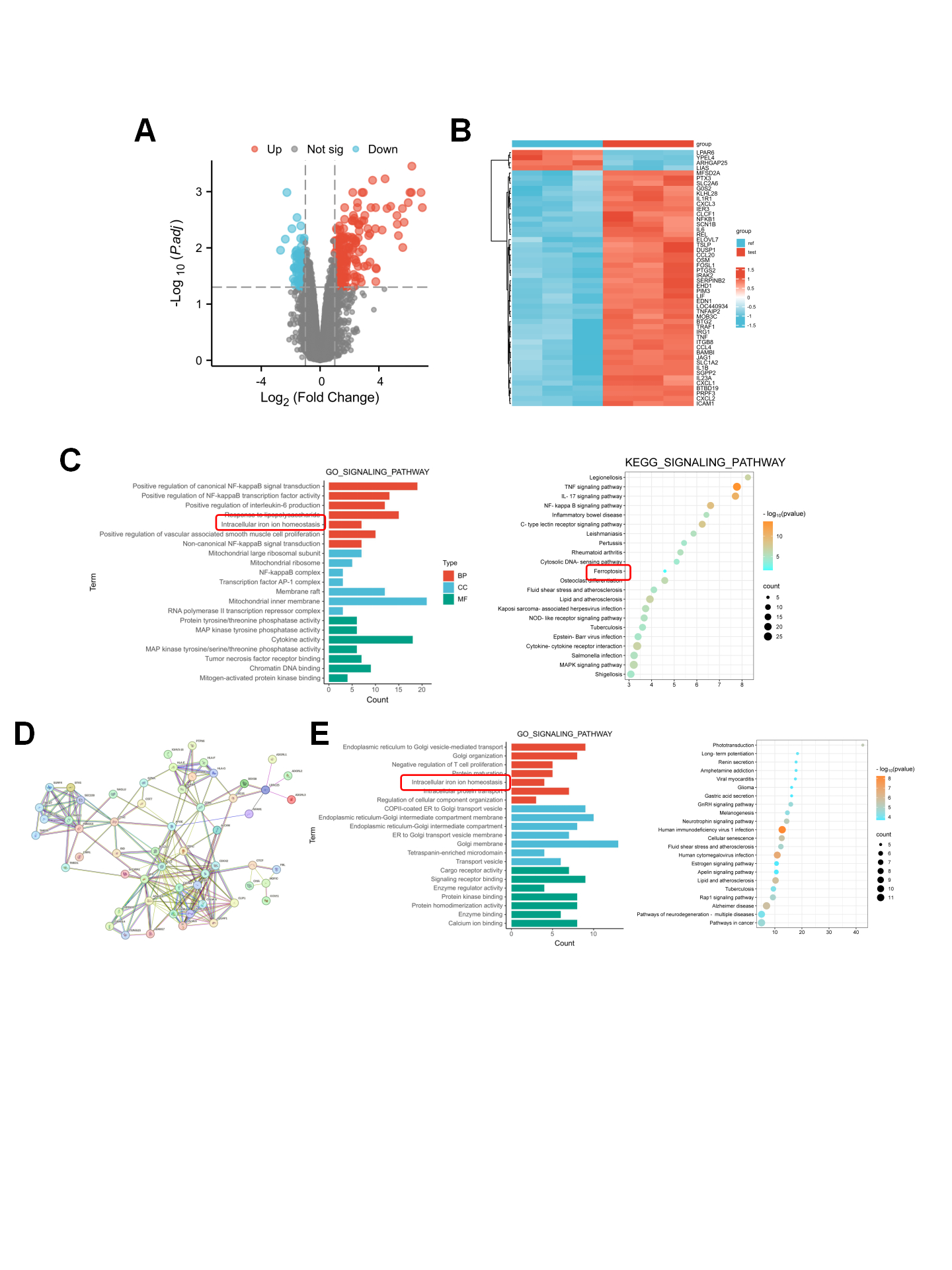
**

**Figure S9**: Expression and enrichment pathways of differentially expressed genes in periodontitis macrophages. (A) Volcano plot illustrating differential gene expression between periodontitis-affected macrophages and control macrophages groups. ﻿(B) The heatmap displays some differentially expressed genes between macrophages affected by periodontitis and the control macrophage group.﻿ (C) Bar plot showing the enrichment analysis of GO signaling pathways associated with the differentially expressed genes. Bubble Chart showing the enrichment analysis of KEGG signaling pathways associated with the differentially expressed genes. The enriched pathways are predominantly related to iron homeostasis, underscoring the potential role of these genes in modulating iron balance in the context of periodontitis. ﻿(D) Network diagram of protein-protein interactions (PPI) among the seven key genes and their associated proteins. The nodes represent proteins, and the edges indicate interactions. ﻿(E) Bar plot depicting the KEGG signaling pathways enrichment analysis for the PPI network proteins. Bubble Chart showing the GO signaling pathways enrichment analysis for the PPI network proteins.
